# Hints for Improvements

**Published:** 1800-10

**Authors:** 


### *Hints for Improvements.*

Phyſicians in all ages have ſaid, that in epilepſy the brain, or a particular ſtate of the nerves, tranſmitted this affection to other parts, and were the primary ſeats of theſe diſorders; and, in fact, this has been demonſtrated from pathological phenomena, by ſeveral anatomiſts and obſervers. Thoſe epilepſies which have their ſeat immediately in the brain, have been found the moſt difficult to cure, but thoſe which have ariſen in the trunk or extremities, have been more eaſily removed. For theſe latter ſpecies, Monſ. Portal, an eminent and reſpectable practitioner in Paris, is of opinion that topical applications produce very beneficial effects, and mentions ſeveral caſes which he had cured, by rubbing the part affected with the *Oleum Animale de Dippel, (Ol. Cornu Cervi rectificat.)* after having uſed the whole tribe of nervous medicines, with bark, zinc, opium, and bliſters, in vain. In another caſe, where a convulſive epileptic affection was attended with conſiderable pain in the right foot, which had been much injured by a fall; both the pain and the epilepſy were ſpeedily removed by rubbing the part with a ſolution of opium in the *Ol. Animale de Dippel.*—This application might be tried in locked jaws.

---

An Eſſay on Medical and Chirurgical Education, by Mr. Parkinson, of Hoxton Square, Author of the Chemical Pocket Book, Medical Admonitions, &c. we underſtand is in the preſs, and will be publiſhed at the meeting of the Claſſes of the different Lectures, to be entitled The Pupil, and is propoſed to contain, 1. Remarks on the qualifications required for thoſe intended for Medical Students; 2. A mode of education propoſed inſtead of that by apprenticeſhip; 3. Hints addreſſed to Hoſpital Pupils; 4. Advice to young men in their firſt eſtabliſhment in the Profeſſion.

---

